# Induction of USP17 by combining BET and HDAC inhibitors in breast cancer cells

**DOI:** 10.18632/oncotarget.5601

**Published:** 2015-09-10

**Authors:** Gabor Borbely, Lars-Arne Haldosen, Karin Dahlman-Wright, Chunyan Zhao

**Affiliations:** ^1^ Karolinska Institutet, Department of Biosciences and Nutrition, Novum, Huddinge, Sweden; ^2^ SciLifeLab, Karolinska Institutet, Solna, Sweden; ^3^ Current address: Swetox & Karolinska Institutet, Unit for Toxicology Sciences, Södertälje, Sweden

**Keywords:** breast cancer, epigenetics, BET, HDAC, combined treatment

## Abstract

Members of the bromodomain and extra-C terminal (BET) domain protein family and the histone deacetylase (HDAC) enzyme family regulate the expression of important oncogenes and tumor suppressor genes. Here we show that the BET inhibitor JQ1 inhibits proliferation and induces apoptosis of both triple negative and estrogen receptor positive breast cancer cells. Consistent with the critical role of histone acetylation in the regulation of gene expression, treatment with JQ1 or the HDAC inhibitor mocetinostat was associated with global changes in gene expression resulting in suppression of genes involved in cell-cycle regulation. Combining JQ1 with mocetinostat, further decreased cell viability. This synergistic effect was associated with increased suppression of genes essential for cell-cycle progression. Furthermore, we detected dramatic increase in the expression of several members of the ubiquitin–specific protease 17 (USP17) family of deubiquitinating enzymes in response to the combination treatment. Increased expression of USP17 enzymes were able to attenuate the Ras/MAPK pathway causing decrease in cell viability, while, siRNA mediated depletion of USP17 significantly decreased cytotoxicity after the combination treatment. In conclusion, our study demonstrates that co-treatment with BET inhibitors and HDAC inhibitors reduces breast cancer cell viability through induction of USP17.

## INTRODUCTION

Breast cancer has the highest incidence and mortality (13%) among all cancer types diagnosed in women worldwide with nearly 1,5 million new cases per year [1]. Histology and biomarkers are tools to subgroup breast cancer and also determine treatment regimen. Estrogen receptor alpha (ER)-expressing breast tumors are classically treated with anti-estrogens. However, some of these tumors present as endocrine resistant at diagnosis or develop resistance during treatment. Treatment of triple-negative breast cancer (TNBC), a tumor type defined by lack of expression of ER, progesterone receptor (PR) and epidermal growth factor receptor 2 (HER2), is a challenge as target-specific drugs are not available [2]. Thus the use of novel molecularly targeted approaches to treat breast cancer is of great interest.

Next to the accumulation of genetic mutations, there is growing evidence that reversible modifications of histone proteins and DNA are crucial to the onset and progression of breast cancer. These, so called epigenetic modifications, play major roles in the regulation and modulation of gene expression, representing an attractive mechanism that can be exploited in breast cancer therapy [3].

Acetylation and deacetylation are among the most abundant histone post-translational modifications and are regulated by two families of enzymes: histone acetyltransferases (HATs) and histone deacetylases (HDACs). HATs facilitate the transfer of acetyl groups to the ε-amino group on lysine residues of histone proteins. HDACs oppose the action of HATs and remove the acetyl group from lysine residues. Acetylation has the effect of neutralizing the overall negative charge of the histone tail. This results in a relaxed structure of the chromatin, permissive to the recruitment of the transcriptional machinery. Significant correlations have been reported between histone modification status, tumor biomarker phenotype, and clinical outcome, where high relative levels of global histone acetylation were associated with a favorable prognosis [4]. HDAC inhibitors (HDACis) are currently considered as candidate new drugs in breast cancer therapy. A number of early phase clinical trials have been completed or are ongoing [3, 5], but the initial optimism has not been completely translated to clinical success. As single agents, HDACis have proven less successful for the treatment of breast cancer therefore, much effort is invested in evaluating rational combinations [3].

Histone acetylation also directly promotes transcription by providing binding sites for BET proteins that recognize or “read” histone modifications and assemble a complex of co-regulatory proteins such as positive transcription elongation factor b complex (P-TEFb) to facilitate gene transcription [6, 7]. The BET family consists of four members in humans, the bromodomain-containing proteins (BRD), BRD2, BRD3 and BRD4 and the bromodomain testis-specific protein (BRDT). Through the initiation of transcription elongation, BRD4 has been shown to increase the transcription of various genes such as MYC, cyclin A, cyclin D1 and BCL2, all involved in initiation and growth of tumors [8, 9, 10].

Several selective, small-molecule BET protein inhibitors have recently been developed. Among these, JQ1 is in preclinical development for cancer treatment as several studies demonstrated its anti-tumorigenic activities in preclinical models of hematological malignancies and neuroblastoma [9, 10, 11]. JQ1 binds competitively to bromodomains to displace BET proteins along with associated transcription factors, from chromatin. Inhibition of BRD4-promoter interactions has been shown to suppress the expression of important cell growth and survival genes, resulting in cell cycle arrest and extensive apoptosis in ER+ breast cancer [12, 13], leukemia [9] and lymphoma [14] cells. However, the utility of BET inhibitors in TNBC has not been investigated.

In this study we show that the small-molecule BET inhibitor JQ1 exerts growth inhibitory effects on a representative panel of TNBC and ER+ breast cancer cell lines and that this is associated with global changes in gene expression. Consistent with a synergistic effect of diverse levels of epigenetic regulation on gene expression, we report synergistic effects of JQ1 and the HDACi mocetinostat on regulation of gene expression. These effects coincided with a significant suppression of several cell-cycle related genes, up-regulation of members of the USP17 family of deubiquitinating enzymes and down-regulation of the Ras/MAP kinase signaling pathway which is commonly dysregulated in TNBC [15].

## RESULTS

### JQ1 exerts pro-apoptotic and anti-proliferative effects in TNBC and ER+ breast cancer cell lines

**Figure 1:**
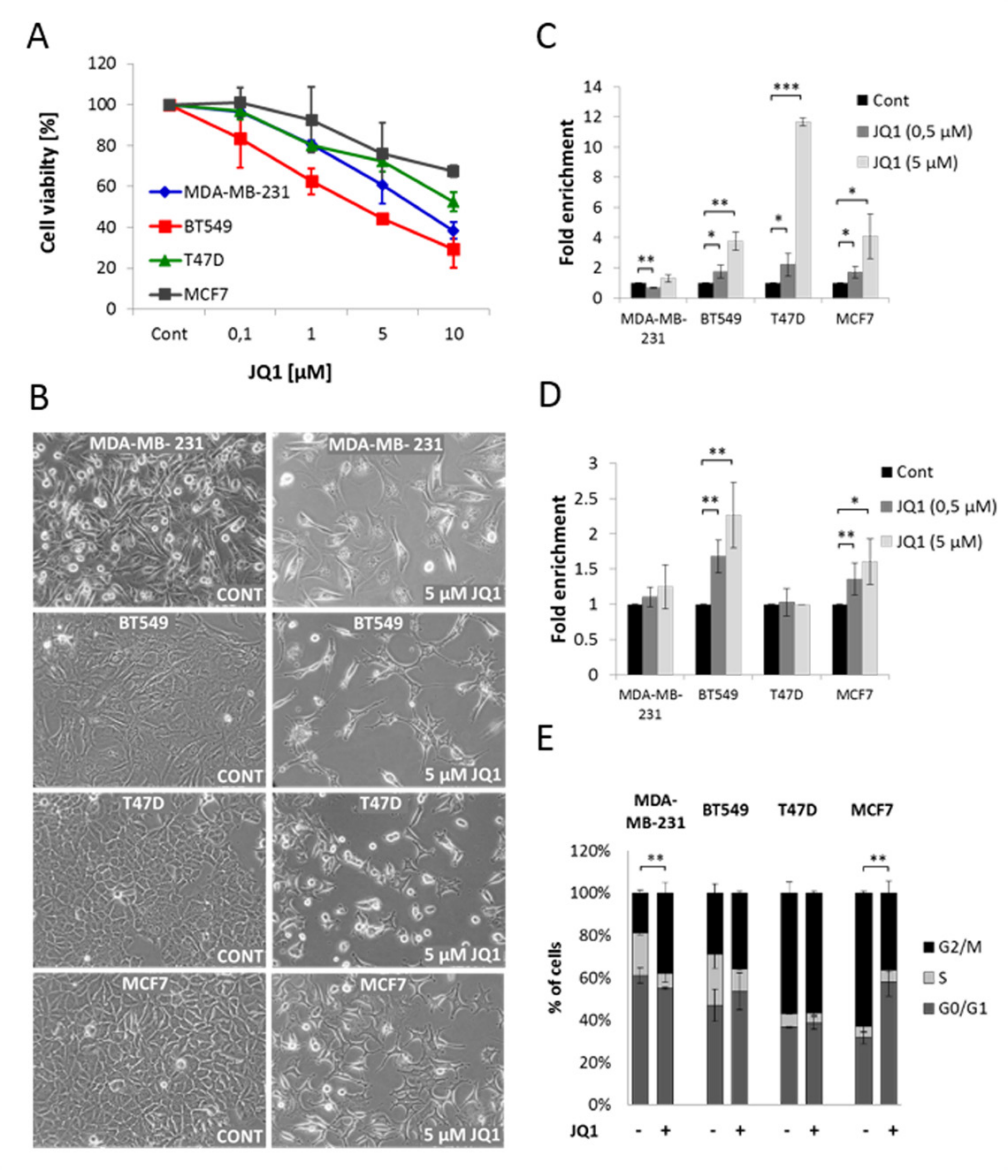
JQ1 decreases cell viability in both TNBC and ER+ breast cancer cell lines **A.** TNBC (MDA-MB-231, BT549) and ER+ (T47D, MCF7) breast cancer cell lines were treated with the indicated concentrations of JQ1 for 48 hours. Changes in cell viability were assayed by the WST-1 cell viability assay. Data are presented as mean (*n* = 3) percentage +/− standard deviation (SD) relative to control. **B.** Visual appearance of MDA-MB-231, BT549, T47D and MCF7 cells following 48 hours treatment with DMSO (control) or 5 μM JQ1. Magnification: 20x. (**C.** and **D.**) MDA-MB-231, BT549, T47D and MCF7 cells were treated with the indicated concentrations of JQ1 for 48 hours. After treatment, JQ1-induced enrichment of nucleosomes in the cytoplasm of cells **C.** and in the culture-supernatant **D.** was measured by an ELISA assay. Data are presented as mean percentage +/− SD relative to control. **E.** Analysis of cell cycle distribution of MDA-MB-231, BT549, T47D and MCF7 cells after 48 hours treatment with 1 μM JQ1. The cell cycle was assayed using PI staining followed by FACS analysis. Error bars represent SD from *n* ≥ 3 independent experiments. Significance (*P* value) indicates the difference in percentage of cells in G2/M or G0/G1 respectively between control and JQ1 treated samples. P value of results in C, D interactions and E was calculated using a two tailed t test (*P < 0.05; **P < 0.01; ***P < 0.001).

To test the effect of JQ1 on breast cancer cells, two TNBC (MDA-MB-231 and BT549) and two ER+ (MCF7 and T47D) breast cancer cell lines were treated with increasing concentrations of JQ1 for 48 hours. Cell viability was determined by the WST-1 assay. We observed that JQ1 significantly decreased cell viability in a dose-dependent manner in all four tested cell lines (Figure 1A). This result was further confirmed by the trypan blue exclusion assay (Figure S1), which serves as an index of cell viability. Visual inspection of cells following JQ1 treatment showed that in addition to a decrease in cell number, cells also changed morphology. These changes were most marked in the BT549 and T47D cell lines which showed various extents of cell shrinkage and cell membrane blebbing consistent with apoptosis (Figure 1B). These observations suggested that JQ1 may induce apoptosis. This was measured by an enzyme-linked immunosorbent assay (ELISA) and flow cytometry. As shown in Figure 1C, JQ1 treatment induced apoptosis in BT549, MCF7 and T47D cells but not in MDA-MB-231 cells. Furthermore, there is an induction of necrosis in the BT549 and MCF7 cells (Figure 1D). Next we used flow cytometry subsequent to staining with propidium iodide (PI) to assess cell cycle progression after 48 hours of JQ1 treatment. The cell cycle analysis revealed that JQ1 treatment induced significant G0/G1 phase and G2/M phase cell cycle arrest in MCF7 and MDA-MB-231 cells respectively (Figure 1E). We did not observe significant changes in the G0/G1 and G2/M transitions in the BT549 and T47D cell lines but JQ1 significantly decreased the S-phase population in all cell lines except MCF7 cells. Together these findings demonstrate that JQ1 exerted significant growth inhibitory effects in the investigated breast cancer cell lines as a result of prolonged duration of the cell cycle and/or increased cell death. JQ1 seems to display cell line selectivity as it does not exert a strong pro-apoptotic activity in MDA-MB-231 cells but exerts anti-proliferative effects at different phases of the cell cycle progression in all cell lines. Finally, JQ1 exerts both pro-apoptotic and anti-proliferative effect in the MCF7 cell line.

### JQ1 attenuates expression of c-Myc in TNBC and ER+ breast cancer cell lines

**Figure 2:**
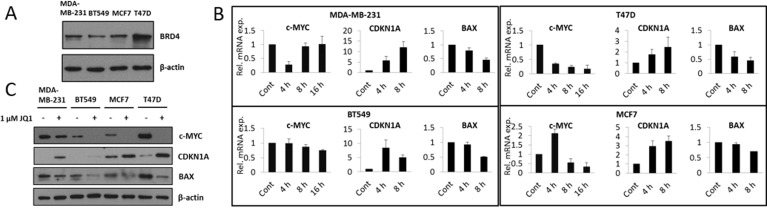
JQ1 treatment attenuates c-Myc expression resulting in increased expression of CDKN1A and decreased expression of BAX, at both the mRNA and protein levels **A.** Total cell lysates were prepared and immunoblot analyses were performed for the detection of BRD4 expression in MDA-MB-231, BT549, MCF7 and T47D breast cancer cell lines. β-actin was used as a loading control. **B.** MDA-MB-231, BT549, MCF7 and T47D cells were treated with 1 μM JQ1 for 4, 8 and 16 hours. Total mRNA was harvested, reverse transcribed, and QPCR was performed for c-Myc, CDKN1A and BAX. mRNA expression is shown relative to the DMSO treated (vehicle) control. Error bars represent SD from three independent experiments. **C.** MDA-MB-231, BT549, MCF7 and T47D cells were treated with 1 μM JQ1 for 48 hours. At the end of the treatment, cells were lysed and analyzed by immunoblot for c-Myc, CDKN1A and BAX protein expression. β-actin was used as a loading control.

It has previously been shown that BRD4 plays an important role in the regulation of cell cycle progression and cell viability. Furthermore, of the BET proteins, BRD4 is the most sensitive to JQ1 treatment [16]. We therefore assessed BRD4 expression in the investigated breast cancer cell lines. BRD4 was found to be expressed in all four cell lines (Figure 2A). BRD4 is known to positively regulate the transcription of c-Myc through the recruitment of P-TEFb, which activates RNA POLII [9]. Consistent with this, JQ1 treatment suppressed c-Myc mRNA expression (Figure 2B). However, the time course was different for the different cell lines. In the MDA-MB-231 cell line we observed a transient down-regulation at the earliest investigated time point (4 hours) after JQ1 treatment. In the BT549 and T47D cell lines, we observed a time dependent decrease in c-Myc mRNA expression, however of different magnitudes. Finally, in the MCF7 cell line, we observed increased c-Myc mRNA expression at an early time point (4 hours) which was followed by a decrease at later time points (8 and 16 hours). Importantly, JQ1 decreased the levels of the c-Myc protein for all cell lines (Figure 2C). c-Myc promotes either cell cycle progression or apoptosis through inhibiting expression of target genes such as CDKN1A, known to inhibit proliferation and inducing expression of pro-apoptotic genes such as BAX [17]. In concert with the attenuation of c-Myc expression, JQ1 treatment up-regulated the mRNA expression of CDKN1A and down-regulated the mRNA expression of BAX (Figure 2B). Similar results were observed at the level of protein expression. JQ1 treatment decreased BAX protein levels and increased CDKN1A protein levels in all four cell lines (Figure 2C).

### Combination treatment with HDAC inhibitors and JQ1 has synergistic effects in breast cancer cell lines

**Figure 3:**
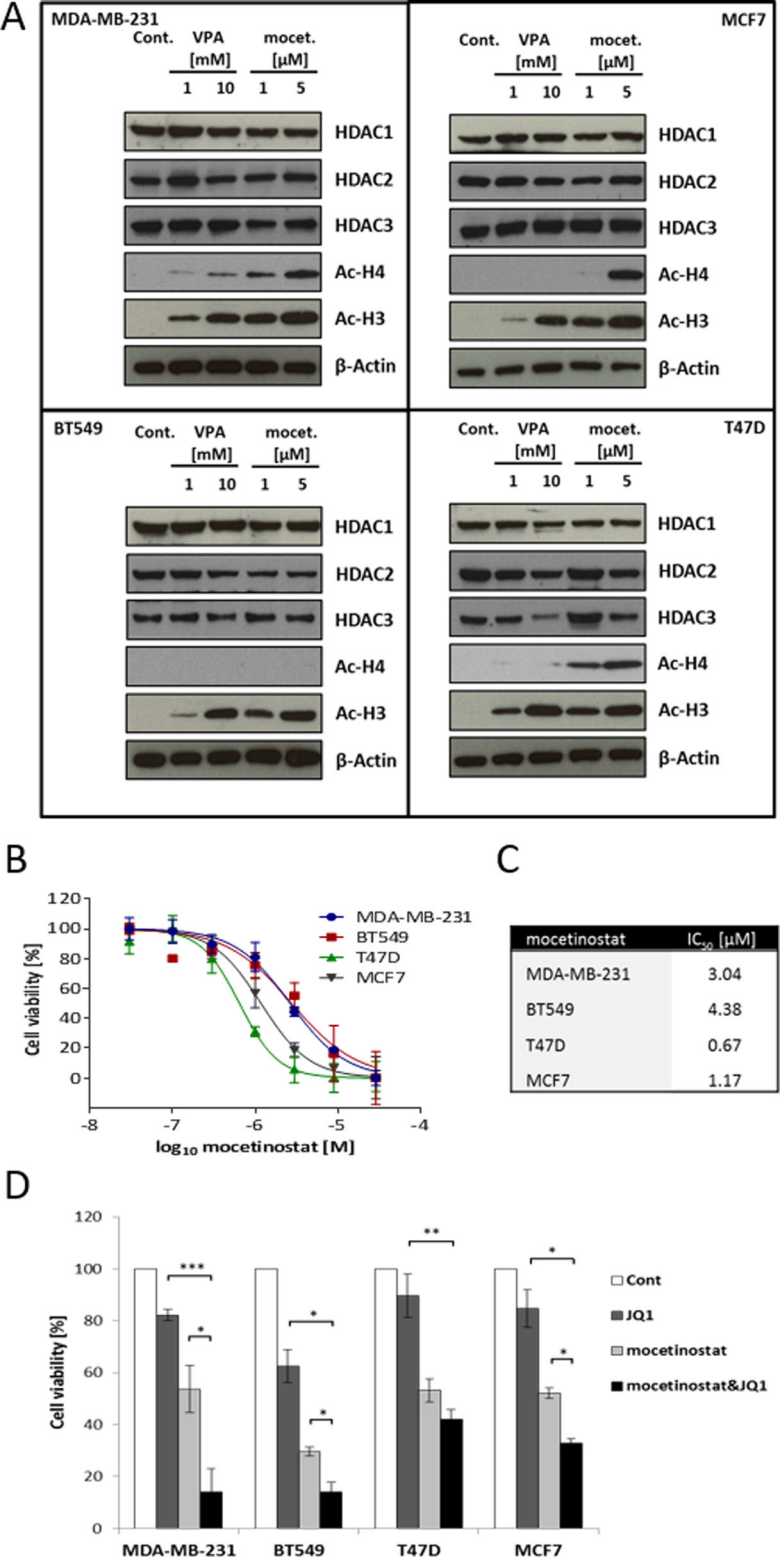
VPA and mocetinostat increase histone H3 and H4 acetylation and reduce cell viability, an effect that is further potentiated by JQ1 treatment **A.** MDA-MB-231, BT549, MCF7 and T47D cells were treated with the indicated concentrations of VPA or mocetinostat for 48 hours. Total protein lysates were analyzed by immunoblot using the indicated antibodies. β-actin was used as a loading control. **B.** Cells were treated with increasing concentrations of mocetinostat for 48 hours and assayed by the WST-1 cell viability assay. **C.** IC_50_ values were calculated by the GraphPad Prism software. **D.** Cells were treated with JQ1 (1 μM) and mocetinostat (3 μM for MDA-MB-231, 4.4 μM for BT549, 0.7 μM for T47D, 1.2 μM for MCF7) for 48 hours and then assayed by the WST-1 cell viability assay. Error bars represent SD from *n* ≥ 3 independent experiments. Significance (P value) of results in B and D was calculated using a two tailed t test (*P < 0.05; **P < 0.01; ***P < 0.001).

To test the efficacy of HDACis on HDAC expression and histone acetylation, the breast cancer cell lines were treated with increasing concentrations of the HDACis, VPA and mocetinostat, independently, for two days. De-acetylation of histone H3 was efficiently inhibited by both mocetinostat and VPA in all four cell lines (Figure 3A). With regard to histone H4, mocetinostat clearly induced hyper-acetylation in all cell lines except BT549. On the contrary, VPA only clearly induced hyper-acetylation in the MDA-MB-231 cell line. None of the HDACis significantly changed the expression of HDAC1, HDAC2 and HDAC3 proteins in MDA-MB-231, BT549 or MCF7 cells but both inhibitors reduced the expression levels of HDAC1, HDAC2 and HDAC3 in T47D cells (Figure 3A). To determine the effect of HDACis on cell viability, the four breast cancer cell lines were treated with increasing concentrations of mocetinostat or VPA for two days. As shown in Figure 3B, mocetinostat induced a significant and dose-dependent decrease in cell viability. Based on the IC_50_ values shown in Figure 3C, ER+ cells were more sensitive to mocetinostat treatment with IC_50_ values of 1.17 μM and 0.67 μM for MCF7 and T47D cells, respectively, compared to IC_50_ values of 4.38 μM and 3.04 μM determined for the TNBC cell lines BT549 and MDA-MB-231, respectively. VPA also decreased cell viability at higher millimolar concentrations (Figure S2A and B).

Since both BET and HDAC inhibitors are known to alter gene expression and both groups of inhibitors attenuated cell viability in our experiments (Figure 1A, 3B and S2A), we next asked if co-treatment with JQ1 and HDACi would further decrease cell viability. All four breast cancer cell lines were treated for two days with a combination of JQ1 (1 μM) and mocetinostat (cell line dependent IC_50_ value) (Figure 3D) or a combination of JQ1 (1 μM) and VPA (cell line dependent IC_50_ value) (Figure S2C). We observed that the combination treatments significantly decreased cell viability compared to treatment with JQ1, mocetinostat or VPA alone in MDA-MB-231, BT549 and MCF7 cell lines but not in the T47D cell line. The results also showed that TNBC cells were more sensitive to both mocetinostat-JQ1 and VPA-JQ1 combinations, as compared to the ER+ cells.

### JQ1 and mocetinostat down-regulate similar cellular pathways

**Figure 4:**
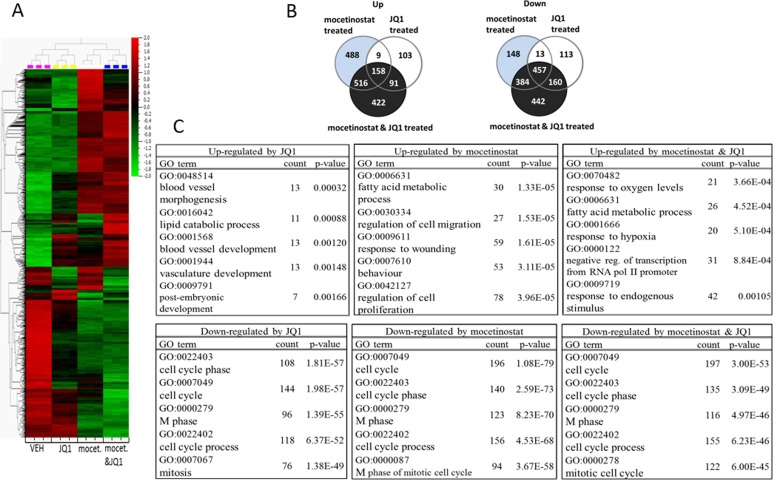
JQ1 and mocetinostat regulate similar cellular pathways mainly involved in cell proliferation, survival and cell cycle progression MDA-MB-231 cells were treated with 1 μM JQ1 or 3 μM mocetinostat or with the combination of both for 48 hours. Total RNA was harvested, reverse transcribed and analyzed for gene expression by the Affymetrix^®^ Human Gene 2.1 ST Array. Modulation of gene expression was determined by comparison of vehicle (DMSO) treated samples. **A.** Heat map showing top 1000 genes that are differently expressed in MDA-MB-231 cells after 48 hours treatment with 1 μM JQ1 or 3 μM mocetinostat or with the combination. Hierarchical clustering was generated using Qlucore Omics Explorer. Red (signifies up-regulation) and green (signifies down-regulation) labels indicate relative gene expression compared to vehicle (DMSO) treated controls. **B.** Venn diagram for genes differentially expressed in MDA-MB-231 breast cancer cells at least 2-fold (*p* < 0.001) following treatment with 1 μM JQ1, 3 μM mocetinostat or with the combination compared with vehicle control. **C.** Top 5 statistically enriched biological process categories affected by JQ1, mocetinostat or the combination treatment evaluated by Database for Annotation, Visualization and Integrated Discovery software (DAVID).

To further characterize the cellular effects of JQ1 and HDACis, alone or in combination, we performed a comparative global gene expression analysis for MDA-MB-231 cells treated with JQ1 or mocetinostat alone or in combination. Transcripts deregulated ≥ 2 fold (P < 0.001) were derived from three independent experiments. The top 1000 up- and down-regulated genes are depicted in the heatmap (Figure 4A). As shown in Figure 4B, a total of 361 and 743 genes were up- and down-regulated, respectively, by JQ1 treatment. The mocetinostat treatment resulted in the up-regulation of 1171 and down-regulation of 1002 genes. The combination treatment up-regulated 1187 genes while the expression of 1443 genes was suppressed.

Global gene expression profiles were further analyzed to reveal possible molecular mechanisms and signaling pathways involved in the response of MDA-MB-231 cells to JQ1, mocetinostat and the combination of these. Figure 4C shows the top five pathways up- and down-regulated by JQ1, mocetinostat or the combination treatment, respectively (Table S3 shows the full list of biological process categories significantly altered by the treatments in MDA-MB-231 cells). The results show that genes down-regulated by the single treatment with JQ1 or mocetinostat or the combination treatment are overlapping and involved in cell cycle regulation, including pathways ‘cell cycle phase’, ‘cell cycle’, ‘M-phase’, ‘cell cycle process’, ‘mitosis’ and ‘M-phase of mitotic cell cycle’. Gene expression levels of well-known cell proliferation promoting genes, e.g. CDK1 (−33-fold), CDCA8 (−32-fold), CCNA2 (−29-fold) were significantly reduced. This result is consistent with our observation of decreased cell cycle progression of MDA-MB-231 cells after treatment with JQ1 alone (Figure 1E).

### Combination treatment with JQ1 and mocetinostat up-regulates USP17 and attenuates the Ras/MAPK signaling pathway, resulting in decreased cell viability

**Figure 5:**
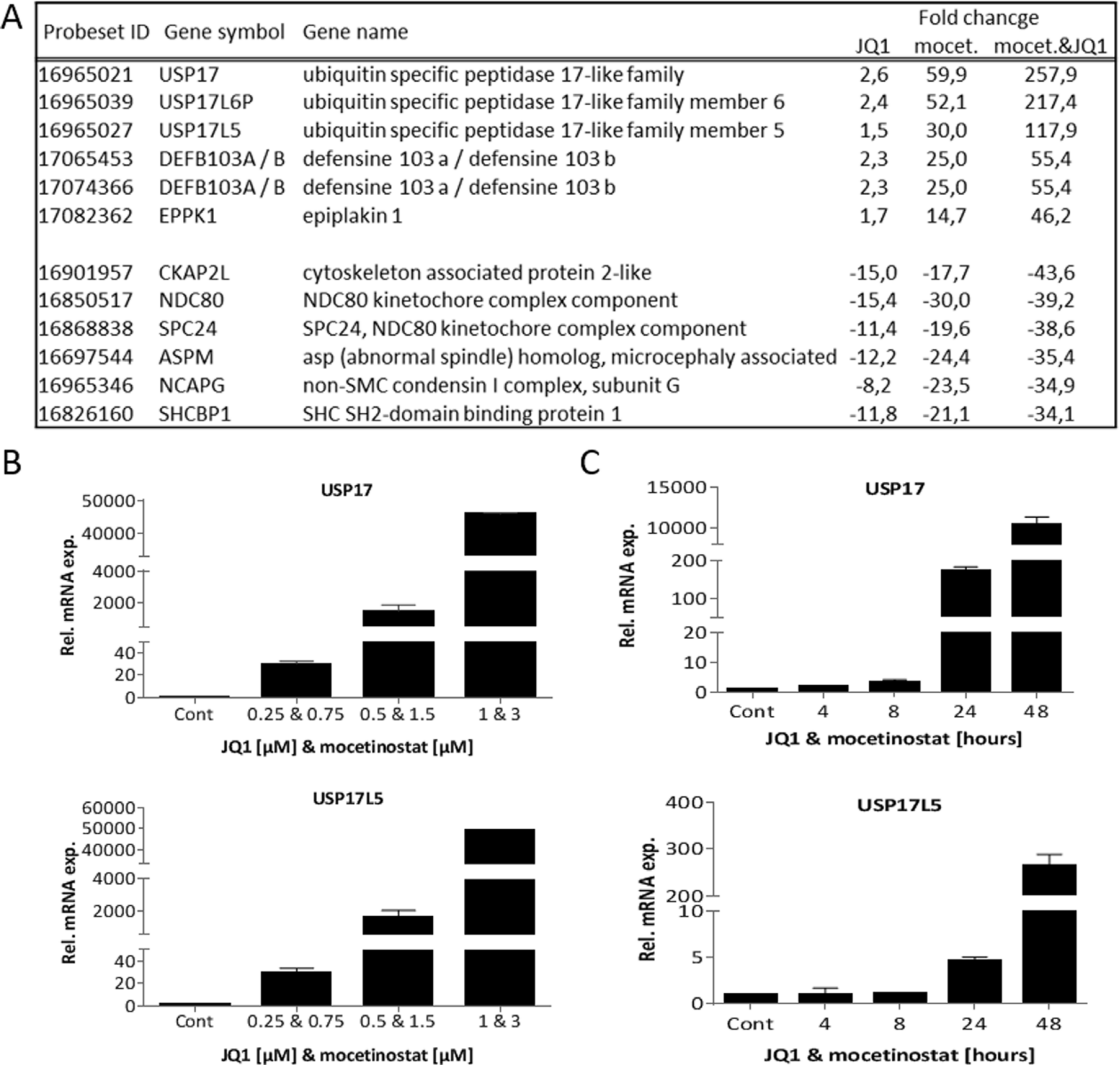
Combination treatment with JQ1 and mocetinostat significantly up-regulates USP17 family members in a time and dose dependent manner **A.** List of the top (ranked by the combination treatment) six up- and down-regulated genes affected by all treatments (JQ1 alone, mocetionsstat alone or the combination treatment). **B.** USP17 and USP17L5 mRNA levels were determined by QPCR in MDA-MB-231 cells after treatment with the indicated concentrations of JQ1 and mocetinostat in combination for 48 hours. **C.** USP17 and USP17L5 mRNA levels were determined by QPCR in MDA-MB-231 cells after a time course of 4-48 hours of JQ1 (1 μM) and mocetinostat (3 μM) combination treatment. mRNA expression is shown relative to the DMSO treated (vehicle) control. Error bars represent SD from *n* ≥ 3 independent experiments.

To reveal molecular mechanisms responsible for the synergistic effect of the combination treatment we focused our attention on the 158 and 457 genes that were up- and down-regulated, respectively, for all treatments. The top six up-regulated and top six down-regulated genes are listed in Figure 5A. Among the top up-regulated genes, we found several members of the USP17 deubiquitinating family that were up-regulated by the single compound treatments and further increased by the combination treatment. Furthermore, cell cycle related genes were down-regulated by the single compound treatment and then further down-regulated by the combination treatment.

**Figure 6:**
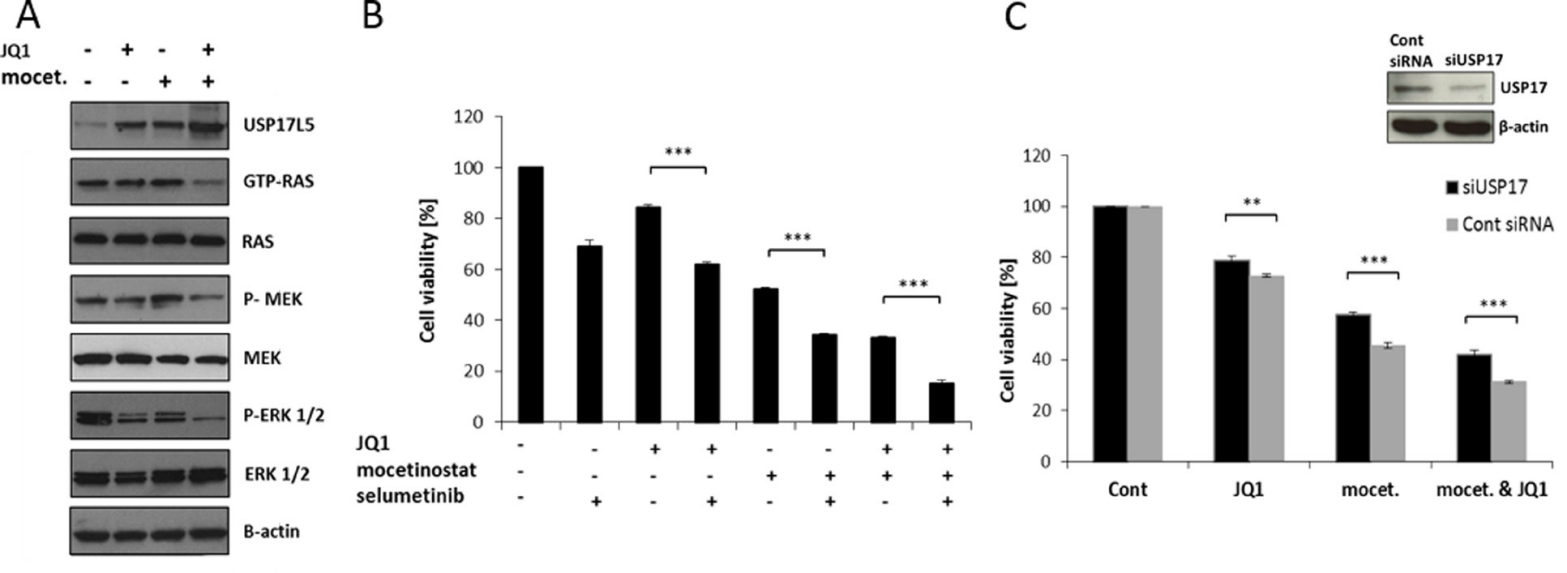
Combination treatment with JQ1 and mocetinostat attenuates the Ras/MEK/ERK pathway through up-regulation of USP17 leading to reduced cell viability **A.** MDA-MB-231 cells were treated with 1 μM JQ1 or 3 μM mocetinostat or with the combination for 48 hours. Ras activity was measured by the pan-Ras activation kit. Total protein lysates were analyzed by immunoblot using antibodies directed against the members of the Ras/MEK/ERK signaling pathway. β-actin was used as a loading control. **B.** Cells were treated with JQ1 (1 μM), mocetinostat (3 μM) and /or selumetinib (10 μM) for 48 hours and then assayed by the WST-1 cell viability assay. **C.** Cell viability of MDA-MB-231 cells 48 hours after siRNA mediated USP17 knockdown compared with cells transfected with control siRNA. Error bars represent SD from n ≥ 3 independent experiments. Significance (*P* value) of results in B and C was calculated using a two tailed t test (*P < 0.05; **P < 0.01; ***P < 0.001). Western blot analysis confirms knockdown of USP17 protein levels. β-*actin* is shown as *loading control*.

We examined USP17 and USP17L5 expression by QPCR in all four breast cancer cell lines after drug exposure. Increased USP17 and USP17L5 expression was found in both ER+ and TNBC cell lines after combination treatment (Figure S3). We further examined dose-responsive changes of USP17 and USP17L5 expression in MDA-MB-231 cells. A dose dependent increase in USP17 and USP17L5 mRNA levels was observed (Figure 5B). Similar results were observed with combination treatments where we applied increasing concentrations of one inhibitor with constant concentration of the other inhibitor (Figure S4). We also examined USP17 and USP17L5 expression at various time points up to 48 hours. The increase in USP17 and USP17L5 mRNA expression was time dependent with a dramatic increase between the 24 and 48 hours time points (Figure 5C). It is well established that USP enzymes regulate cell growth and survival [18]. Furthermore, USP17 inhibits cell proliferation [19] partly through the regulation of the activity of the Ras converting enzyme 1 (RCE1) resulting in a decrease in activity of the Ras/MAPK signaling pathway [20], which could also explain our results shown in Figure 1E. To confirm if the dramatic increase in USP17 mRNA expression was associated with a corresponding decrease in the activity of Ras signaling, we treated MDA-MB-231 cells with 1 μM JQ1 or 3 μM mocetinostat or with their combination for two days and examined the protein expression level of one member of the USP17 family (USP17L5) and also the activity of the Ras/MAPK signaling pathway. Figure 6A shows a significant increase of the USP17L5 protein level in MDA-MB-231 cells after treatment with JQ1 or mocetinostat. The USP17L5 protein expression was further increased by the combination treatment. We found the levels of activated Ras were markedly reduced in samples treated with the combination of JQ1 and mocetinostat. Consistent with the lower level of activated Ras, levels of phosphorylated MEK and ERK1/2 were markedly down-regulated.

To support that blockade of the Ras/MAPK pathway is involved in the synergistic effect of the combination treatment, we used selumetinib to specifically block the MEK protein. MDA-MB-231 cells were treated with JQ1, mocetinostat or selumetinib alone or with their combinations. Selumetinib treatment led to a significant decrease in cell viability in all combinations compared to single agent treatment (Figure 6B). We next determined whether siRNA-mediated USP17 depletion would affect the cytotoxicity following drug treatment. We observed that knockdown of USP17 significantly increased the cell viability after 48 hours treatment with JQ1 (1 μM) or mocetinostat (3 μM) or with their combination (Figure 6C).

## DISCUSSION

It has previously been demonstrated that BET inhibitor treatment leads to cell cycle arrest and apoptosis in different cancer forms such as acute myelogenous leukemia, medulloblastoma and Burkitt's lymphoma [9, 10, 16, 21]. The BET inhibitor JQ1 increased the percentage of cells in the G1-phase and reduced the percentage of cells in the S-phase in leukemia cells [9]. In this study we demonstrated that JQ1 can influence proliferation and apoptosis in breast cancer cell lines representative of two different types of breast cancer. We utilized MDA-MB-231 and BT549 cell lines as models of TNBC and MCF7 and T47D cell lines as models of ER+ breast cancer. We further provided evidence that JQ1, dose-dependently decreased cell viability in all four breast cancer cell lines (Figure 1A). We also demonstrated that JQ1 displays diverse cell line dependent growth inhibitory actions showing mainly pro-apoptotic effects in T47D and BT549 cell lines, purely anti-proliferative effects in the MDA-MB-231 cell line and both pro-apoptotic and anti-proliferative effects in the MCF7 cell line (Figure 1B-1E). Similarly, effects of JQ1 with regard to progression through the cell cycle, also displayed cell line selectivity. JQ1 efficiently attenuated both c-Myc mRNA and protein expression in all four breast cancer cell lines (Figure 2B, 2C). Knowing the central role of c-Myc in proliferation and malignant transformation of human and animal cells [22] and the growth inhibitory effect of JQ1, it is tempting to conclude that targeting the BET-family of proteins represents an exciting novel approach to treat breast cancer. However, the complexity of c-Myc regulation and the differences in cellular response to JQ1 treatment also underline the importance of further characterizing the effects of JQ1 treatment on c-Myc signaling in different *in vitro* and *in vivo* models of breast cancer.

Several reports demonstrate a critical role of HDACs in epigenetic regulation of gene expression in breast cancer [23]. Besides romidepsin and vorinostat, which have been approved by the US Food and Drug Administration for the treatment of cutaneous T-cell lymphoma, an increasing number of HDACis have been developed and recently advanced to clinical trials [24]. Many HDACis have demonstrated preclinical efficacy as monotherapy for hematological malignancies however, as single agents they have proven less successful for the treatment of solid tumor malignancies [3]. Therefore, much effort has been invested in evaluating rational combinations of HDACis and interest in HDACis in combination therapy is growing.

Selective targeting of epigenetic readers as a potential therapeutic strategy became available recently with the recent discovery of BET bromodomain inhibitors such as JQ1 [25], I-BET151 [26], I-BET726 [27]. Previous studies in hematological malignancies have suggested that co-treatment with BET and HDAC inhibitors is more effective than each agent alone [9]. We tested two HDACis different in their structural characteristics, selectivity, efficacy and clinical developmental phase. VPA is a broad-spectrum HDACi with relatively weak (millimolar) inhibition on HDACs [28] with a clear advantage that it has been on the market for non-oncological uses for decades. Mocetinostat is a benzamide HDACi, selective of HDAC1, 2 and 3 enzymes [29]. In accordance with previous reports, we found that both HDACis induce hyperacetylation of histones H3 and H4 and decrease cell viability in all four tested cell lines (Figure 3A-3C, S2A, B). We tested both VPA and mocetinostat in combination with JQ1 and both combinations decreased cell viability synergistically in the MDA-MB-231, BT549 and MCF7 cell lines but not in the T47D cell line (Figure 3D, S2C). Since single agent treatment with JQ1, VPA or mocetinostat was comparable and equally efficient in all four cell lines, the lack of significant synergistic effects in the T47D cell line requires further investigation.

TNBC, lacking ER, PR and HER2 is considered to constitute the most drug-resistant and difficult-to-treat subtypes of breast cancers [2, 15]. The MDA-MB-231 cell line is representing the mesenchymal-like (ML), claudin-low subtype of TNBC which lacks luminal differentiation markers, but shows high enrichment of genes involved in cell motility, epithelial to mesenchymal transition (EMT) and growth factor signalling pathways among others [30]. Overall, patients with claudin-low tumors have an increased likelihood of distant recurrence and death partly due to the highly invasive nature of the disease [18, 31]. We chose the MD-MB-231 cell line for a more extensive molecular characterization as it displayed extensive decrease in cell viability after the combination treatment and we prioritized to further examine this highly aggressive and treatment resistant subtype of breast cancer. Since mocetinostat is active at low micromolar concentrations (Figure 3C) and induced a higher level of histone acetylation especially on histone H4 (Figure 3A), we chose the mocetinostat-JQ1 combination treatment to investigate global gene expression changes in MDA-MB-231 cells upon treatment. Gene expression profiling revealed that both JQ1 and mocetinostat had extensive and similar effects on the transcriptome (Figure 4A, 4B). Furthermore, gene ontology analysis showed that both monotherapy and combination treatment affected genes involved in cell cycle progression and proliferation (Figure 4C and S3). We also observed significant time- and dose-dependent increases in the expression of several members of the USP17 subfamily of cytokine-inducible deubiquitinating enzymes (Figure 5A-5C).

DUB enzymes constitute a large family of proteases, essential in the renewal of the polyubiquitin chains for use during ubiquitination. USP is one of the five sub-families of DUB enzymes. Several lines of evidence suggest that the USP17 family regulates cell growth and survival and that constitutive expression of USP17 can block cell proliferation [18, 32]. Furthermore, USP17-mediated Lys-63-specific deubiquitination of SDS3 (a key component of the HDAC-dependent Sin3A co-repressor complex) resulted in decreased activity of HDACs in HeLa cells [33]. This raises the possibility that a positive feedback loop may exist between USP17 and HDACs which leads to a further decrease of HDAC activity following up-regulation of USP17.

It has also been reported that USP17 is capable of regulating Ras/MAPK signaling partly through the regulation of the RCE1 [18]. Since Ras is a key proto-oncogene in a number of cancers and the aberrant regulation of the Ras/MAPK pathway is one of the most common events in breast cancer progression [15] we tested the activity of the Ras/MAPK pathway upon JQ1 and mocetinostat single agent treatments and co-treatment. We observed an increase in USP17L5 protein expression and a decrease in the activity of Ras (GTP-bound Ras) after combination treatment (Figure 6A). In accordance with decreased Ras activation, we also found decreased phosphorylation levels of MEK and ERK1/2 enzymes after combination treatment, meanwhile none of the tested components of the Ras/MAPK pathway (including Ras, MEK and ERK1/2) showed altered protein expression levels in response to the treatments. Furthermore, substitution for each drug with the selective MEK1/2 inhibitor, selumetinib resulted in decreased cell viability (Figure 6B) while siRNA mediated silencing of USP17 rescued MDA-MB-231 cells from either JQ1 or mocetinostat single agent or combination treatments (Figure 6C), suggesting that the Ras/MAPK signaling pathway is involved in the synergistic effect of the combination treatment.

In summary, we have shown that the BET bromodomain inhibitor JQ1 decreased cell viability in cultured human breast cancer cells representing both TNBC and ER+ breast cancers. Our finding also revealed that JQ1 treatment potentiated the anti-proliferative and pro-apoptotic effects of the HDACis VPA and mocetinostat. Gene expression profiling revealed that both JQ1 and mocetinostat induced similar changes in the transcriptome of MDA-MB-231 cells which resulted in down-regulation of cell cycle related genes and up-regulation of several members of the USP17 sub-family. This latter effect could likely be connected to the stronger anti-proliferative effect of the combination treatment through the down-regulation of the Ras/MAPK signaling pathway. Our study demonstrates that co-treatment with BET inhibitors and HDAC inhibitors reduces breast cancer cell viability through induction of USP17, suggesting that such a regimen may be an effective treatment for breast cancer.

## MATERIALS AND METHODS

### Cell lines and reagents

Human breast cancer cell lines (MDA-MB-231, BT549, MCF7 and T47D) were obtained from the American Type Culture Collection (ATCC, Manassas, VA, USA). MCF7 and T47D cell lines are characterized as representing ER+, luminal A breast cancer and BT549 and MDA-MB-231 cell lines as triple negative, basal B breast cancer. MCF7 and MDA-MB-231 cells were routinely cultured at 37°C, 5% CO_2_ in Dulbecco's Modified Eagle's medium (DMEM) (Invitrogen, Carlsbad, CA, USA) supplemented with 10% heat-inactivated fetal bovine serum (FBS) (Invitrogen), 1% penicillin/streptomycin. T47D and BT549 cells were cultured under the same conditions but in Roswell Park Memorial Institute (RPMI) (Invitrogen) medium supplemented with 10% heat-inactivated FBS and 1% penicillin/streptomycin. Valproic acid (Sigma-Aldrich, St Louis, MO, USA) was dissolved in sterile water at a stock concentration of 0.4 M. MGCD-0103 (mocetinostat) (SelleckChem, Houston, TX, USA), (S)-JQ1 (Abcam, Cambridge, UK) and AZD6244 (selumetinib) (SelleckChem) were prepared as 1 mM stocks in 100% dimethyl sulfoxide. All stocks were frozen at −80°C in 20 μl aliquots.

### WST-1 cell viability assay

Cell viability was assessed using the WST-1 assay (Roche, Mannheim, Germany) following the manufacturer's instructions. Cells cultured in 96 well plates at a concentration of 8 × 10^3^ cells/well and incubated in the presence of compounds in complete medium for the indicated times. Three hours after the addition of WST-1, absorbance was measured at 440 nm and 650 nm (as reference wavelength) using a TECAN Infinite® 200 PRO multimode reader (TECAN, Maennedorf, Switzerland).

### Trypan blue exclusion assay

Trypan blue solution (0.4%)(Invitrogen), is used as a cell stain to assess cell viability using the dye exclusion test. MCF7, T47D, BT549 and MDA-MB-231 cells were seeded at 3×10^5^ cells/well in six-well plates. Following 48 hours treatment with inhibitors or vehicle, cells were trypsinized and cell suspensions mixed with trypan blue stain (0.4%) at a one to one ratio. Cells were counted using Countess Automated Cell Counter (Invitrogen).

### Enzyme-linked immunosorbent assay (ELISA) for detection of cell death

MCF7, T47D, BT549 and MDA-MB-231 cells were plated in 96-well tissue culture plates at 1 × 10^4^ cells/well and treated with the indicated concentrations of JQ1 for 48 hours. Apoptosis and necrosis, were determined with the Cell Death Detection ELISA PLUS Assay kit (Roche) as previously described [34] in accordance with the manufacturer's instructions.

### Flow cytometry

For cell-cycle analysis, cells were harvested by trypsinization, washed twice in phosphate-buffered saline (PBS, pH 7.4), then fixed in 70% ice-cold ethanol and stored on ice for at least 1 hour. Before analysis, the fixed cells were incubated with 10 μg/ml propidium iodide (Sigma-Aldrich) and 100 μg/ml DNase free ribonuclease A (Sigma-Aldrich) for 30 min. The cell-cycle phase distribution was analyzed using FACSCalibur flow cytometer (BD biosciences, Franklin Lakes, NJ, USA).

### Immunoblot analysis

Immunoblotting was performed using standard protocols using c-MYC, p21, Ac-H3 (Lys9/Lys14), Ac-H4 (Lys5), p-MEK, MEK, p-ERK1/2, ERK1/2 (Cell Signaling, Danvers, MA, USA), BAX, HDAC1, HDAC2, HDAC3 (Santa Cruz Inc., Dallas, TX, USA), BRD4 (Bethyl Laboratories Inc., Montgomery, TX, USA), beta-actin (Sigma-Aldrich), USP17L5 (Abcam) primary antibodies and horse radish peroxidase (HRP) conjugated anti-mouse IgG or anti-rabbit IgG (GE Healthcare, Piscataway, NJ, USA) secondary antibodies.

### RNA isolation and quantitative PCR

Total RNA was isolated with the RNeasy Mini Kit (Qiagen, Hilden, Germany) according to the manufacturer's instructions and reverse transcribed from 1 μg RNA. Quantitative real time PCR (QPCR) analysis for the expression of c-MYC, CDKN1A and BAX was carried out with SYBR-Green PCR Master Mix (Applied Biosystems, Foster City, CA, USA) in an ABI PRISM 7500 apparatus (Applied Biosystems) with the following primers: 36B4 forward: 5′-GTGTTCGACAATGGCAGCAT-3′, reverse: 5′-GACACCCTCCAGGAAGCGA-3; c-MYC forward: 5′-GAGCCCCTGGTGCTCCAT-3′, reverse: 5′-TCATCTTCTTGTTCCTCCTCAGAGT-3′; CDKN1A forward: 5′-AGGTGGACCTGGAGACTCTCAG-3′, reverse: 5′-TCCTCTTGGAGAAGATCAGCCG-3′; BAX forward: 5′-CCCGAGAGGTCTTTTTCCGAG-3′, reverse: 5′-CCAGCCCATGATGGTTCTGAT-3′. All target gene transcripts were normalized to the expression of ribosomal phosphoprotein, 36B4. The optimum concentration of primers was determined in preliminary experiments and all primer pairs were tested with melting curves.

### Gene expression microarray analysis

Total RNA was extracted using the RNeasy Mini Kit (Qiagen). Samples from three independent biological replicates for each treatment were hybridized to the Affymetrix Human Gene 2.1 ST array. Target synthesis and hybridizations were performed by the Bioinformatic and Expression Analysis core facility (BEA, www.bea.ki.se, Novum, Karolinska Institutet, Huddinge, Sweden) according to standard protocols. We applied a filter of P < 0.001 for significantly modulated gene expression and at least a 2.0-fold change in mean differential expression. Gene ontology analysis was carried out using DAVID tools. The expression microarray data have been deposited in the GEO database under accession number GSE65495.

### Selective inhibition of USP17 expression by short interfering RNA (siRNA)

USP17 siRNA and nonspecific control siRNA were purchased from Santa Cruz Inc. MDA-MB-231 cells were plated at 2.5 × 10^5^ cells/well in 6 well plates. Double-stranded siRNAs were transfected into MDA-MB-231 cells using the INTERFERin transfection kit (Polyplus transfection Inc., Illkirch, France) according to the manufacturer's instructions. 24 hours after transfection, cells were subjected to Western blot analysis or treatment and WST-1 cell viability assay.

### Pan-Ras activation assay

Pan-Ras activation was measured by a pan-Ras activation kit (Cell Biolabs, San Diego, CA, USA) according to the manufacturer's protocol. Briefly, MDA-MB-231 cells were cultured in 6 cm cell culture dishes in the presence or absence of inhibitors for 48 hours. After treatment, the cells were immediately washed with ice-cold PBS and lysed in lysis/assay buffer containing 125 mM HEPES, pH 7.5, 750 mM NaCl, 5% NP-40, 50 mM MgCl2, 5 mM EDTA, 10% glycerol, supplemented with protease inhibitor cocktail (Roche). Lysates were kept on ice for 15 min and spun down at 13000 g for 10 min. Supernatants were added to Raf1 RBD (Ras-binding domain) Agarose beads to selectively isolate and pull-down the active forms of Ras (GTP-bound Ras). The pulldowns were separated and immunoblotted as previously described (REF) using Anti-pan-Ras antibody (Cell Biolabs).

## SUPPLEMENTARY MATERIAL FIGURES AND TABLE



## References

[1] Jemal A, Bray F, Center MM, Ferlay J, Ward E, Forman D (2011). Global cancer statistics. CA. Cancer J. Clin.

[2] Mataga MA, Rosenthal S, Heerboth S, Devalapalli A, Kokolus S, Evans LR (2012). Anti-breast cancer effects of histone deacetylase inhibitors and calpain inhibitor. Anticancer Res.

[3] Thurn KT, Thomas S, Moore A, Munster PN (2011). Rational therapeutic combinations with histone deacetylase inhibitors for the treatment of cancer. Future Oncol.

[4] Elsheikh SE, Green AR, Rakha EA, Powe DG, Ahmed RA, Collins HM, Soria D, Garibaldi JM, Paish CE, Ammar AA, Grainge MJ, Ball GR, Abdelghany MK (2009). Global histone modifications in breast cancer correlate with tumor phenotypes, prognostic factors, and patient outcome. Cancer Res.

[5] Siegel D, Hussein M, Belani C, Robert F, Galanis E, Richon VM, Garcia-Vargas J, Sanz-Rodriguez C, Rizvi S (2009). Vorinostat in solid and hematologic malignancies. J. Hematol. Oncol.

[6] Patel MC, Debrosse M, Smith M, Dey A, Huynh W, Sarai N, Heightman TD, Tamura T, Ozato K (2013). BRD4 coordinates recruitment of pause release factor P-TEFb and the pausing complex NELF/DSIF to regulate transcription elongation of interferon-stimulated genes. Mol. Cell Biol.

[7] Arrowsmith CH, Bountra C, Fish PV, Lee K, Schapira M (2012). Epigenetic protein families: a new frontier for drug discovery. Nat. Rev. Drug Discov.

[8] Garnier JM, Sharp PP, Burns CJ (2013). BET bromodomain inhibitors: a patent review. Expert Opin. Ther. Pat.

[9] Fiskus W, Sharma S, Qi J, Valenta JA, Schaub LJ, Shah B, Peth K, Portier BP, Rodriguez M, Devaraj SG, Zhan M, Sheng J, Iyer SP (2014). Highly active combination of BRD4 antagonist and histone deacetylase inhibitor against human acute myelogenous leukemia cells. Mol. Cancer. Ther.

[10] Henssen A, Thor T, Odersky A, Heukamp L, El-Hindy N, Beckers A, Speleman F, Althoff K, Schäfers S, Schramm A, Sure U, Fleischhack G, Eggert A (2013). BET bromodomain protein inhibition is a therapeutic option for medulloblastoma. Oncotarget.

[11] Molenaar JJ, Domingo-Fernández R, Ebus ME, Lindner S, Koster J, Drabek K, Mestdagh P, van Sluis P, Valentijn LJ, van Nes J, Broekmans M, Haneveld F, Volckmann R (2012). LIN28B induces neuroblastoma and enhances MYCN levels via let-7 suppression. Nat. Genet.

[12] Sengupta S, Biarnes MC, Clarke R, Jordan VC (2015). Inhibition of BET proteins impairs estrogen-mediated growth and transcription in breast cancers by pausing RNA polymerase advancement. Breast Cancer Res. Treat.

[13] Bihani T, Ezell SA, Ladd B, Grosskurth SE, Mazzola AM, Pietras M, Reimer C, Zinda M, Fawell S, D'Cruz CM (2015). Resistance to everolimus driven by epigenetic regulation of MYC in ER+ breast cancers. Oncotarget.

[14] Bhadury J, Nilsson LM, Muralidharan SV, Green LC, Li Z, Gesner EM, Hansen HC, Keller UB, McLure KG, Nilsson JA (2014). BET and HDAC inhibitors induce similar genes and biological effects and synergize to kill in Myc-induced murine lymphoma. PNAS.

[15] Giltnane JM, Balko JM (2014). Rationale for targeting the Ras/MAPK pathway in triple-negative breast cancer. Discov. Med.

[16] Mertz JA, Conery AR, Bryant BM, Sandy P, Balasubramanian S, Mele DA, Bergeron L, Sims RJ (2011). Targeting MYC dependence in cancer by inhibiting BET bromodomains. Proc. Natl. Acad. Sci. USA.

[17] Xu J, Chen Y, Olopade OI (2010). MYC and Breast Cancer. Genes Cancer.

[18] Burrows JF, Kelvin AA, McFarlane C, Burden RE, McGrattan MJ, De la Vega M, Govender U, Quinn DJ, Dib K, Gadina M, Scott CJ, Johnston JA (2009). USP17 regulates Ras activation and cell proliferation by blocking RCE1 activity. J. Biol. Chem.

[19] Ramakrishna S, Suresh B, Bae SM, Ahn WS, Lim KH, Baek KH (2012). Hyaluronan binding motifs of USP17 and SDS3 exhibit anti-tumor activity. PLoS One.

[20] Burrows JF, Scott CJ, Johnston JA (2010). The DUB/USP17 deubiquitinating enzymes: a gene family within a tandemly repeated sequence, is also embedded within the copy number variable beta-defensin cluster. BMC Genomics.

[21] Delmore JE, Issa GC, Lemieux ME, Rahl PB, Shi J, Jacobs HM, Kastritis E, Gilpatrick T, Paranal RM, Qi J, Chesi M, Schinzel AC, McKeown MR (2011). BET bromodomain inhibition as a therapeutic strategy to target c-Myc. Cell.

[22] Liao DJ, Dickson RB (2000). c-Myc in breast cancer. Endocr. Relat. Cancer.

[23] Ropero S, Esteller M (2007). The role of histone deacetylases (HDACs) in human cancer. Mol. Oncol.

[24] Connolly R, Stearns V (2012). Epigenetics as a therapeutic target in breast cancer. J. Mammary Gland Biol. Neoplasia.

[25] Sanchez R, Zhou MM (2009). The role of human bromodomains in chromatin biology and gene transcription. Curr. Opin. Drug Discov. Devel.

[26] Seal J, Lamotte Y, Donche F, Bouillot A, Mirguet O, Gellibert F, Nicodeme E, Krysa G, Kirilovsky J, Beinke S, McCleary S, Rioja I, Bamborough P (2012). Identification of a novel series of BET family bromodomain inhibitors: binding mode and profile of I-BET151 (GSK1210151A). Bioorg. Med. Chem. Lett.

[27] Gosmini R, Nguyen VL, Toum J, Simon C, Brusq JM, Krysa G, Mirguet O, Riou-Eymard AM, Boursier EV, Trottet L, Bamborough P, Clark H, Chung CW (2014). The discovery of I-BET726 (GSK1324726A), a potent tetrahydroquinoline ApoA1 up-regulator and selective BET bromodomain inhibitor. J. Med. Chem.

[28] Göttlicher M, Minucci S, Zhu P, Krämer OH, Schimpf A, Giavara S, Sleeman JP, Lo Coco F, Nervi C, Pelicci PG, Heinzel T (2001). Valproic acid defines a novel class of HDAC inhibitors inducing differentiation of transformed cells. EMBO J.

[29] Fournel M, Bonfils C, Hou Y, Yan PT, Trachy-Bourget MC, Kalita A, Liu J, Lu AH, Zhou NZ, Robert MF, Gillespie J, Wang JJ, Ste-Croix H (2008). MGCD0103, a novel isotype-selective histone deacetylase inhibitor, has broad spectrum antitumor activity *in vitro* and *in vivo*. Mol. Cancer Ther.

[30] Lehmann BD, Bauer JA, Chen X, Sanders ME, Chakravarthy AB, Shyr Y, Pietenpol JA (2011). Identification of human triple-negative breast cancer subtypes and preclinical models for selection of targeted therapies. J. Clin. Invest.

[31] Prat A, Parker JS, Karginova O, Fan C, Livasy C, Herschkowitz JI, He X, Perou CM (2010). Phenotypic and molecular characterization of the claudin-low intrinsic subtype of breast cancer. Breast Cancer Res.

[32] Burrows JF, McGrattan MJ, Johnston JA (2005). The DUB/USP17 deubiquitinating enzymes, a multigene family within a tandemly repeated sequence. Genomics.

[33] Ramakrishna S, Suresh B, Lee EJ, Lee HJ, Ahn WS, Baek KH (2011). Lys-63-specific Deubiquitination of SDS3 by USP17 Regulates HDAC Activity. J Biol Chem.

[34] Balijepalli MK, Tandra S, Pichika MR (2010). Antiproliferative activity and induction of apoptosis in estrogen receptor-positive and negative human breast carcinoma cell lines by Gmelina asiatica roots. Pharmacognosy. Res.

