# Supplementary material for: Induction of USP17 by combining BET and HDAC inhibitors in breast cancer cells
Source: Oncotarget. 2015 Sep 10;6(32):33623–35. doi: 10.18632/oncotarget.5601 (PMC4741790; doi:10.18632/oncotarget.5601)
Supplement: Supplementary file 1 [file oncotarget-06-33623-s001.pdf]

Induction of USP17 by combining BET and HDAC inhibitors in breast cancer cells

Supplementary Material

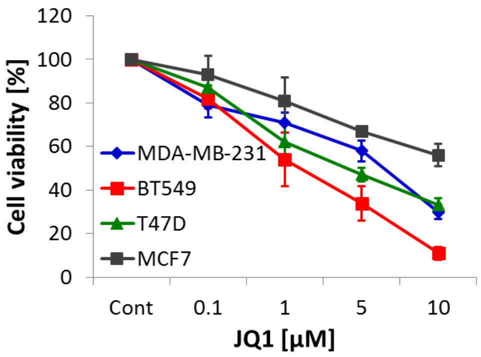

Figure S1. JQ1 decreases cell viability of both TNBC and ER+ breast cancer cell lines. TNBC (MDA-MB-231, BT549) and ER+ (T47D, MCF7) breast cancer cell lines were treated with the indicated concentrations of JQ1 for 48 hours. Changes in cell viability were assayed by trypan blue exclusion assay. Data are presented as mean (n=3) percentage +/- standard deviation (SD) relative to control.

A

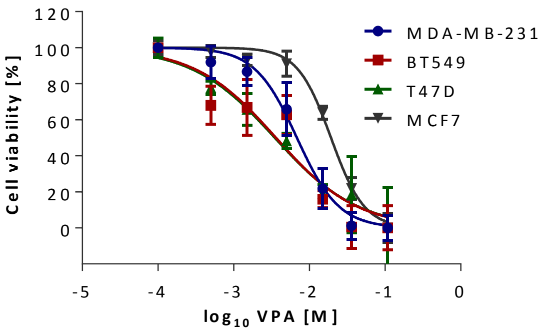

B

VPA	IC_{50} [mM]
MDA-MB-231	8.06
BT549	9.02
T47D	4.01
MCF7	21.36

C

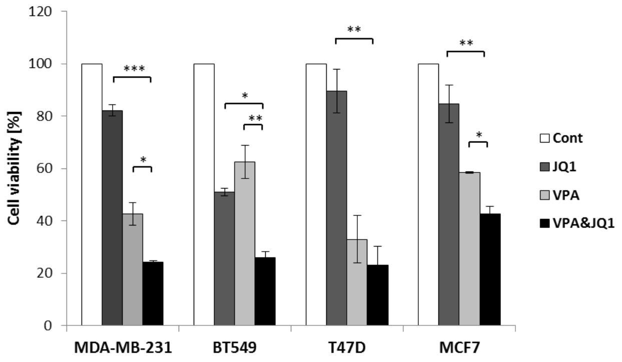

Figure S2. VPA reduces cell viability which is further potentiated by JQ1 treatment. (A) MDA-MB-231, BT549, T47D and MCF7 cells were treated with increasing concentrations of VPA for 48 hours and assayed by WST-1 cell viability assay. (**B**) IC_{50} values were calculated by GraphPad Prism software. (**C**) Cells were treated with JQ1 (1 μM) and VPA (8 mM for MDA-MB-231, 9 mM for BT549, 4 mM for T47D, 21.4 mM for MCF7) for 48 hours then assayed by WST-1 cell viability assay. Error bars represent SD from n ≥ 3 independent experiments. Significance (P value) of results in A and C was calculated using a two tailed t test (* P<0.05; ** P<0.01; *** P<0.001).

Table S1.

Full list of statistically enriched biological process categories affected by JQ1 (**A, B**), mocetinostat (**C, D**) or their combination (**E, F**) treatment evaluated by Database for Annotation, Visualization and Integrated Discovery (DAVID) software.

A

Down-regulated by JQ1		
GO term	count	p-alue
GO:0022403~cell cycle phase	108	1.81E-57
GO:0007049~cell cycle	144	1.98E-57
GO:0000279~M phase	96	1.39E-55
GO:0022402~cell cycle process	118	6.37E-52
GO:0007067~mitosis	76	1.38E-49
GO:0000280~nuclear division	76	1.38E-49
GO:0000087~M phase of mitotic cell cycle	76	6.20E-49
GO:0048285~organelle fission	76	3.84E-48
GO:0000278~mitotic cell cycle	93	1.26E-47
GO:0051301~cell division	77	1.96E-40
GO:0007059~chromosome segregation	36	1.08E-27
GO:0006259~DNA metabolic process	75	1.29E-22
GO:0006260~DNA replication	45	1.13E-21
GO:0051726~regulation of cell cycle	56	1.42E-19
GO:0000070~mitotic sister chromatid segregation	20	1.12E-17
GO:0000819~sister chromatid segregation	20	2.22E-17
GO:0006974~response to DNA damage stimulus	52	1.56E-14
GO:0006281~DNA repair	43	2.53E-13
GO:0007346~regulation of mitotic cell cycle	31	3.80E-13
GO:0000075~cell cycle checkpoint	24	9.58E-13
GO:0010564~regulation of cell cycle process	26	3.07E-12
GO:0051327~M phase of meiotic cell cycle	23	3.83E-11
GO:0007126~meiosis	23	3.83E-11
GO:0007017~microtubule-based process	37	4.23E-11
GO:0051321~meiotic cell cycle	23	5.86E-11
GO:0051276~chromosome organization	53	1.17E-10
GO:0007051~spindle organization	16	1.20E-10
GO:0000226~microtubule cytoskeleton organization	26	9.94E-10
GO:0033554~cellular response to stress	56	1.33E-09
GO:0006323~DNA packaging	23	1.43E-09
GO:0051329~interphase of mitotic cell cycle	21	4.49E-09
GO:0006261~DNA-dependent DNA replication	16	6.37E-09
GO:0051325~interphase	21	7.55E-09
GO:0007093~mitotic cell cycle checkpoint	14	8.21E-09
GO:0008283~cell proliferation	43	1.66E-07
GO:0007076~mitotic chromosome condensation	8	2.39E-07
GO:0007088~regulation of mitosis	14	2.62E-07
GO:0051783~regulation of nuclear division	14	2.62E-07
GO:0030261~chromosome condensation	10	3.14E-07
GO:0006955~immune response	54	5.18E-06
GO:0048015~phosphoinositide-mediated signaling	15	1.10E-05
GO:0042325~regulation of phosphorylation	40	1.43E-05

Down-regulated by JQ1		
GO term	count	p-value
GO:0051303~establishment of chromosome localization	7	1.57E-05
GO:0050000~chromosome localization	7	1.57E-05
GO:0065004~protein-DNA complex assembly	15	1.64E-05
GO:0006270~DNA replication initiation	7	2.43E-05
GO:0019220~regulation of phosphate metabolic process	40	3.51E-05
GO:0051174~regulation of phosphorus metabolic process	40	3.51E-05
GO:0051797~regulation of hair follicle development	5	3.74E-05
GO:0042634~regulation of hair cycle	5	3.74E-05
GO:0007010~cytoskeleton organization	37	4.02E-05
GO:0010948~negative regulation of cell cycle process	8	4.38E-05
GO:0031577~spindle checkpoint	6	6.69E-05
GO:0000725~recombinational repair	7	7.43E-05
GO:0000724~double-strand break repair via homologous recombination	7	7.43E-05
GO:0051640~organelle localization	14	8.13E-05
GO:0042770~DNA damage response, signal transduction	13	8.42E-05
GO:0040001~establishment of mitotic spindle localization	5	1.64E-04
GO:0006302~double-strand break repair	11	1.76E-04
GO:0030071~regulation of mitotic metaphase/anaphase transition	7	1.84E-04
GO:0031497~chromatin assembly	13	1.92E-04
GO:0000910~cytokinesis	9	2.02E-04
GO:0031570~DNA integrity checkpoint	10	2.13E-04
GO:0007052~mitotic spindle organization	6	2.29E-04
GO:0009611~response to wounding	40	2.31E-04
GO:0010604~positive regulation of macromolecule metabolic process	57	2.55E-04
GO:0000079~regulation of cyclin-dependent protein kinase activity	10	2.87E-04
GO:0006310~DNA recombination	14	3.15E-04
GO:0000082~G1/S transition of mitotic cell cycle	10	3.80E-04
GO:0051785~positive regulation of nuclear division	7	3.94E-04
GO:0045840~positive regulation of mitosis	7	3.94E-04
GO:0031399~regulation of protein modification process	26	4.09E-04
GO:0051656~establishment of organelle localization	11	4.33E-04
GO:0045787~positive regulation of cell cycle	10	4.36E-04
GO:0051653~spindle localization	5	4.60E-04
GO:0051293~establishment of spindle localization	5	4.60E-04
GO:0006334~nucleosome assembly	12	5.54E-04
GO:0019932~second-messenger-mediated signaling	22	5.89E-04
GO:0006333~chromatin assembly or disassembly	15	6.25E-04
GO:0010212~response to ionizing radiation	10	6.43E-04
GO:0007018~microtubule-based movement	14	6.45E-04
GO:0007094~mitotic cell cycle spindle assembly checkpoint	5	7.00E-04
GO:0051310~metaphase plate congression	5	7.00E-04
GO:0045841~negative regulation of mitotic metaphase/anaphase transition	5	7.00E-04

B

Up-regulated by JQ1		
GO term	count	p-value
GO:0048514~blood vessel morphogenesis	13	3.20E-04
GO:0016042~lipid catabolic process	11	8.83E-04

C

Down-regulated by mocetinostat		
GO term	count	p-value
GO:0007049~cell cycle	196	1.08E-79
GO:0022403~cell cycle phase	140	2.59E-73
GO:0000279~M phase	123	8.23E-70
GO:0022402~cell cycle process	156	4.53E-68
GO:0000087~M phase of mitotic cell cycle	94	3.67E-58
GO:0000280~nuclear division	93	7.21E-58
GO:0007067~mitosis	93	7.21E-58
GO:0000278~mitotic cell cycle	117	3.49E-57
GO:0048285~organelle fission	93	5.20E-56
GO:0051301~cell division	97	8.26E-49
GO:0007059~chromosome segregation	44	6.76E-33
GO:0006260~DNA replication	64	1.08E-32
GO:0006259~DNA metabolic process	102	6.54E-31
GO:0051276~chromosome organization	95	9.38E-28
GO:0006974~response to DNA damage stimulus	74	5.98E-22
GO:0051726~regulation of cell cycle	69	9.99E-22
GO:0000070~mitotic sister chromatid segregation	22	7.53E-18
GO:0000819~sister chromatid segregation	22	1.65E-17
GO:0006281~DNA repair	57	3.08E-17
GO:0000226~microtubule cytoskeleton organization	39	5.45E-16
GO:0051321~meiotic cell cycle	32	1.03E-15
GO:0033554~cellular response to stress	82	1.61E-15
GO:0007017~microtubule-based process	51	1.89E-15
GO:0007051~spindle organization	22	3.32E-15
GO:0007126~meiosis	31	4.46E-15
GO:0051327~M phase of meiotic cell cycle	31	4.46E-15
GO:0007346~regulation of mitotic cell cycle	38	1.00E-14
GO:0010564~regulation of cell cycle process	32	5.87E-14
GO:0006261~DNA-dependent DNA replication	22	1.47E-12
GO:0000075~cell cycle checkpoint	27	1.82E-12
GO:0006325~chromatin organization	57	1.18E-11
GO:0051329~interphase of mitotic cell cycle	27	4.01E-11
GO:0051325~interphase	27	8.03E-11
GO:0006323~DNA packaging	28	1.56E-10
GO:0033043~regulation of organelle organization	37	2.84E-09
GO:0007010~cytoskeleton organization	57	2.98E-09
GO:0007093~mitotic cell cycle checkpoint	15	3.94E-08
GO:0008283~cell proliferation	54	5.25E-08
GO:0051052~regulation of DNA metabolic process	24	5.37E-08
GO:0051297~centrosome organization	13	6.15E-08
GO:0006297~nucleotide-excision repair, DNA gap filling	10	7.59E-08
GO:0006333~chromatin assembly or disassembly	25	1.03E-07
GO:0016568~chromatin modification	39	1.53E-07
GO:0006302~double-strand break repair	17	1.65E-07
GO:0031023~microtubule organizing center organization	13	1.95E-07
GO:0051783~regulation of nuclear division	16	2.37E-07
GO:0007088~regulation of mitosis	16	2.37E-07
GO:0050000~chromosome localization	9	3.87E-07
GO:0051303~establishment of chromosome localization	9	3.87E-07
GO:0065004~protein-DNA complex assembly	20	4.40E-07
GO:0006270~DNA replication initiation	9	7.36E-07
GO:0030071~regulation of mitotic metaphase/anaphase transition	10	1.21E-06
GO:0032886~regulation of microtubule-based process	14	1.70E-06
GO:0070507~regulation of microtubule cytoskeleton organization	13	1.80E-06

Down-regulated by mocetinostat		
GO term	count	p-value
GO:0007076~mitotic chromosome condensation	8	1.98E-06
GO:0034508~centromere complex assembly	6	2.94E-06
GO:0031570~DNA integrity checkpoint	14	3.52E-06
GO:0000725~recombinational repair	9	3.73E-06
GO:0000724~double-strand break repair via homologous recombination	9	3.73E-06
GO:0030261~chromosome condensation	10	4.27E-06
GO:0006310~DNA recombination	20	4.28E-06
GO:0010604~positive regulation of macromolecule metabolic process	80	4.32E-06
GO:0000079~regulation of cyclin-dependent protein kinase activity	14	5.55E-06
GO:0042770~DNA damage response, signal transduction	17	6.40E-06
GO:0000910~cytokinesis	12	9.54E-06
GO:0051310~metaphase plate congression	7	1.03E-05
GO:0045787~positive regulation of cell cycle	14	1.05E-05
GO:0034621~cellular macromolecular complex subunit organization	41	1.71E-05
GO:0031497~chromatin assembly	17	1.96E-05
GO:0048015~phosphoinositide-mediated signaling	17	2.27E-05
GO:0006275~regulation of DNA replication	14	2.76E-05
GO:0006289~nucleotide-excision repair	13	3.68E-05
GO:0007098~centrosome cycle	8	4.35E-05
GO:0006338~chromatin remodeling	13	4.45E-05
GO:0000082~G1/S transition of mitotic cell cycle	13	4.45E-05
GO:0034728~nucleosome organization	17	4.61E-05
GO:0006268~DNA unwinding during replication	7	5.78E-05
GO:0007127~meiosis I	11	7.44E-05
GO:0008156~negative regulation of DNA replication	9	9.81E-05
GO:0048477~oogenesis	10	1.78E-04
GO:0010605~negative regulation of macromolecule metabolic process	65	1.92E-04
GO:0006334~nucleosome assembly	15	1.93E-04
GO:0051053~negative regulation of DNA metabolic process	10	2.20E-04
GO:0043933~macromolecular complex subunit organization	63	2.33E-04
GO:0000077~DNA damage checkpoint	11	2.45E-04
GO:0007096~regulation of exit from mitosis	6	2.93E-04
GO:0031577~spindle checkpoint	6	2.93E-04
GO:0032392~DNA geometric change	7	2.95E-04
GO:0032508~DNA duplex unwinding	7	2.95E-04
GO:0051656~establishment of organelle localization	13	3.63E-04
GO:0034622~cellular macromolecular complex assembly	34	3.87E-04
GO:0045449~regulation of transcription	181	4.78E-04
GO:0051640~organelle localization	15	5.08E-04
GO:0080135~regulation of cellular response to stress	16	5.26E-04
GO:0046605~regulation of centrosome cycle	5	5.45E-04
GO:0040001~establishment of mitotic spindle localization	5	5.45E-04
GO:0051172~negative regulation of nitrogen compound metabolic process	48	6.08E-04
GO:0051383~kinetochore organization	4	6.48E-04
GO:0008629~induction of apoptosis by intracellular signals	11	6.63E-04
GO:0042325~regulation of phosphorylation	44	7.03E-04
GO:0045935~positive regulation of nucleic acid metabolic process	55	7.23E-04
GO:0010557~positive regulation of macromolecule biosynthetic process	57	7.27E-04
GO:0045934~negative regulation of nucleic acid metabolic process	47	8.22E-04
GO:0051173~positive regulation of nitrogen compound metabolic process	56	8.93E-04
GO:0000731~DNA synthesis during DNA repair	5	9.39E-04
GO:0007080~mitotic metaphase plate congression	5	9.39E-04
GO:0007052~mitotic spindle organization	6	9.68E-04
GO:0031399~regulation of protein modification process	31	9.73E-04

D

Up-regulated by mocetinostat		
GO term	count	p-value
GO:0006631~fatty acid metabolic process	30	1.33E-05
GO:0030334~regulation of cell migration	27	1.53E-05
GO:0009611~response to wounding	59	1.61E-05
GO:0007610~behavior	53	3.11E-05
GO:0042127~regulation of cell proliferation	78	3.96E-05
GO:0048514~blood vessel morphogenesis	30	4.47E-05
GO:0001568~blood vessel development	33	5.10E-05
GO:0040012~regulation of locomotion	28	5.43E-05
GO:0051270~regulation of cell motion	28	5.95E-05
GO:0001944~vasculature development	33	8.17E-05
GO:0044057~regulation of system process	38	9.12E-05
GO:0045765~regulation of angiogenesis	14	1.06E-04
GO:0051240~positive regulation of multicellular organismal process	32	1.12E-04
GO:0002504~antigen processing	10	1.30E-04
GO:0019229~regulation of vasoconstriction	10	1.67E-04

Up-regulated by mocetinostat		
GO term	count	p-value
GO:0030155~regulation of cell adhesion	21	2.95E-04
GO:0010033~response to organic substance	69	3.25E-04
GO:0070482~response to oxygen levels	21	4.33E-04
GO:0008015~blood circulation	25	5.12E-04
GO:0003013~circulatory system process	25	5.12E-04
GO:0006954~inflammatory response	37	5.14E-04
GO:0046394~carboxylic acid biosynthetic process	22	5.88E-04
GO:0016053~organic acid biosynthetic process	22	5.88E-04
GO:0001666~response to hypoxia	20	5.99E-04
GO:0007167~enzyme linked receptor protein signaling pathway	38	6.77E-04
GO:0009719~response to endogenous stimulus	43	7.29E-04
GO:0051046~regulation of secretion	26	7.38E-04
GO:0045907~positive regulation of vasoconstriction	6	7.67E-04
GO:0001525~angiogenesis	21	8.13E-04
GO:0010035~response to inorganic substance	26	9.16E-04

E

Down-regulated by JQ1 & mocetinostat		
GO term	count	p-value
GO:0007049~cell cycle	197	3.00E-53
GO:0022403~cell cycle phase	135	3.09E-49
GO:0000279~M phase	116	4.97E-46
GO:0022402~cell cycle process	155	6.23E-46
GO:0000278~mitotic cell cycle	122	6.00E-45
GO:0007067~mitosis	91	1.47E-42
GO:0000280~nuclear division	91	1.47E-42
GO:0000087~M phase of mitotic cell cycle	91	8.61E-42
GO:0048285~organelle fission	91	7.36E-41
GO:0051301~cell division	93	2.06E-32
GO:0007059~chromosome segregation	44	1.36E-26
GO:0006260~DNA replication	59	2.58E-20
GO:0006259~DNA metabolic process	104	4.88E-20
GO:0051726~regulation of cell cycle	75	5.39E-17
GO:0006974~response to DNA damage stimulus	80	1.23E-16
GO:0000819~sister chromatid segregation	23	1.28E-15
GO:0000070~mitotic sister chromatid segregation	22	9.51E-15
GO:0033554~cellular response to stress	94	4.31E-12
GO:0007346~regulation of mitotic cell cycle	41	4.95E-12
GO:0051276~chromosome organization	84	7.70E-12
GO:0010564~regulation of cell cycle process	34	2.25E-11
GO:0051329~interphase of mitotic cell cycle	31	1.54E-10
GO:0006281~DNA repair	56	2.87E-10
GO:0007051~spindle organization	20	3.02E-10
GO:0051325~interphase	31	3.36E-10
GO:0000075~cell cycle checkpoint	28	8.05E-10
GO:0007017~microtubule-based process	51	9.03E-10
GO:0000226~microtubule cytoskeleton organization	36	2.02E-09
GO:0051321~meiotic cell cycle	28	7.91E-09
GO:0007126~meiosis	27	2.22E-08
GO:0051327~M phase of meiotic cell cycle	27	2.22E-08
GO:0033043~regulation of organelle organization	43	3.77E-08
GO:0006261~DNA-dependent DNA replication	20	4.38E-08
GO:0008283~cell proliferation	67	1.62E-07
GO:0050000~chromosome localization	10	3.61E-07
GO:0051303~establishment of chromosome localization	10	3.61E-07
GO:0007088~regulation of mitosis	18	7.67E-07
GO:0051783~regulation of nuclear division	18	7.67E-07
GO:0051310~metaphase plate congression	8	4.55E-06
GO:0030261~chromosome condensation	11	9.50E-06
GO:0042770~DNA damage response, signal transduction	20	1.01E-05
GO:0006270~DNA replication initiation	9	1.04E-05
GO:0006323~DNA packaging	25	1.11E-05
GO:0000079~regulation of cyclin-dependent protein kinase activity	16	1.15E-05

Down-regulated by JQ1 & mocetinostat		
GO term	count	p-value
GO:0070507~regulation of microtubule cytoskeleton organization	14	1.21E-05
GO:0032886~regulation of microtubule-based process	15	1.57E-05
GO:0007093~mitotic cell cycle checkpoint	14	1.61E-05
GO:0007076~mitotic chromosome condensation	8	2.05E-05
GO:0030071~regulation of mitotic metaphase/anaphase transition	10	2.16E-05
GO:0006915~apoptosis	77	2.27E-05
GO:0045787~positive regulation of cell cycle	16	2.34E-05
GO:0007010~cytoskeleton organization	60	2.61E-05
GO:0051174~regulation of phosphorus metabolic process	65	2.67E-05
GO:0019220~regulation of phosphate metabolic process	65	2.67E-05
GO:0042325~regulation of phosphorylation	63	2.78E-05
GO:0012501~programmed cell death	77	3.82E-05
GO:0080135~regulation of cellular response to stress	22	4.25E-05
GO:0031399~regulation of protein modification process	44	5.84E-05
GO:0070302~regulation of stress-activated protein kinase signaling pathway	17	6.83E-05
GO:0032270~positive regulation of cellular protein metabolic process	37	6.88E-05
GO:0051247~positive regulation of protein metabolic process	38	7.51E-05
GO:0051439~regulation of ubiquitin-protein ligase activity during mitotic cell cycle	17	9.85E-05
GO:0010627~regulation of protein kinase cascade	38	1.26E-04
GO:0051338~regulation of transferase activity	51	1.27E-04
GO:0010604~positive regulation of macromolecule metabolic process	98	1.40E-04
GO:0045859~regulation of protein kinase activity	48	1.45E-04
GO:0008630~DNA damage response, signal transduction in induction of apoptosis	11	1.50E-04
GO:0032268~regulation of cellular protein metabolic process	61	1.55E-04
GO:0043549~regulation of kinase activity	49	1.70E-04
GO:0008629~induction of apoptosis by intracellular signals	14	2.16E-04
GO:0007080~mitotic metaphase plate congression	6	2.87E-04
GO:0043408~regulation of MAPKKK cascade	21	2.91E-04
GO:0051438~regulation of ubiquitin-protein ligase activity	17	3.14E-04
GO:0000082~G1/S transition of mitotic cell cycle	14	3.19E-04
GO:0000724~double-strand break repair via homologous recombination	8	3.96E-04
GO:0000725~recombinational repair	8	3.96E-04
GO:0046328~regulation of JNK cascade	15	4.35E-04
GO:0051340~regulation of ligase activity	17	4.91E-04
GO:0034508~centromere complex assembly	5	5.02E-04
GO:0007089~traversing start control point of mitotic cell cycle	5	5.02E-04
GO:0031570~DNA integrity checkpoint	13	5.69E-04
GO:0008219~cell death	82	5.87E-04
GO:0051297~centrosome organization	10	5.95E-04
GO:0016265~death	82	7.16E-04
GO:0051640~organelle localization	18	7.43E-04
GO:0006302~double-strand break repair	14	9.02E-04
GO:0051493~regulation of cytoskeleton organization	23	9.08E-04

F

Up-regulated by JQ1 & mocetinostat		
GO term	count	p-value
GO:0070482~response to oxygen levels	21	3.66E-04
GO:0006631~fatty acid metabolic process	26	4.52E-04
GO:0001666~response to hypoxia	20	5.10E-04
GO:0000122~negative regulation of transcription from RNA polymerase II promoter	31	8.84E-04

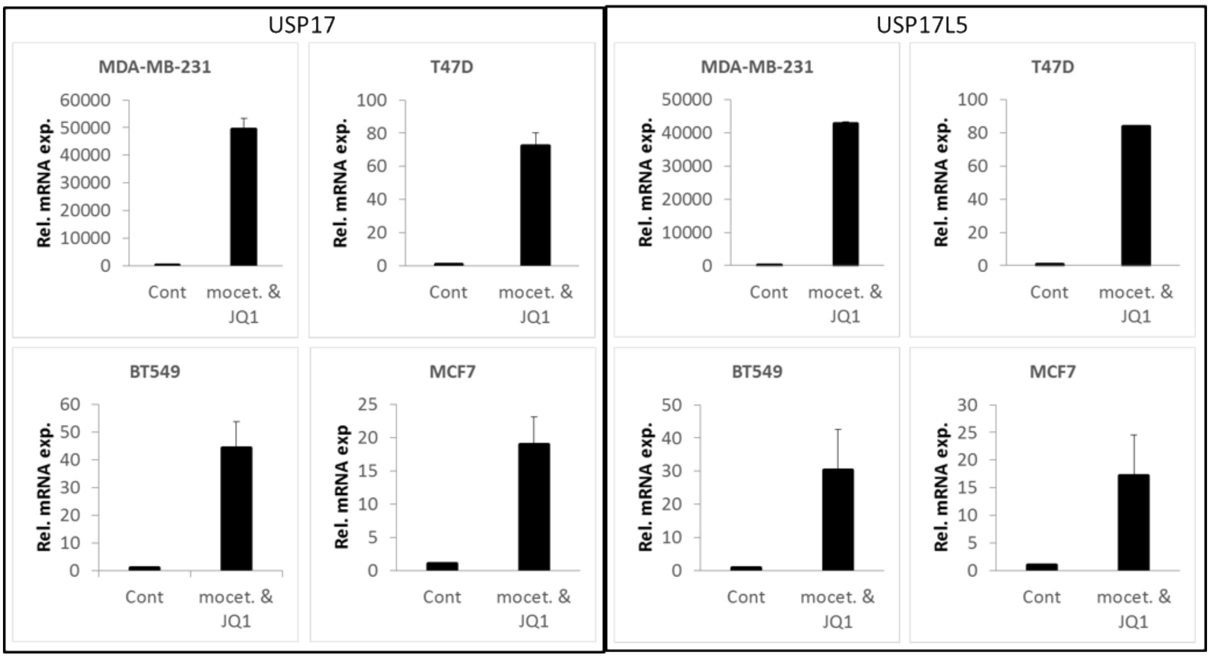

Figure S3. USP17 and USP17L5 mRNA levels were up-regulated in all four (TNBC and ER+) breast cancer cell lines after combination treatment. Cells were treated with JQ1 (1 µM) and mocetinostat (3 µM for MDA-MB-231, 4.4 µM for BT549, 0.7 µM for T47D, 1.2 µM for MCF7) for 48 hours. Total mRNA was harvested, reverse transcribed, and QPCR was performed for USP17 and USP17L5. mRNA expression is shown relative to the DMSO treated (vehicle) control. Error bars represent SD from n ≥ 3 independent experiments.

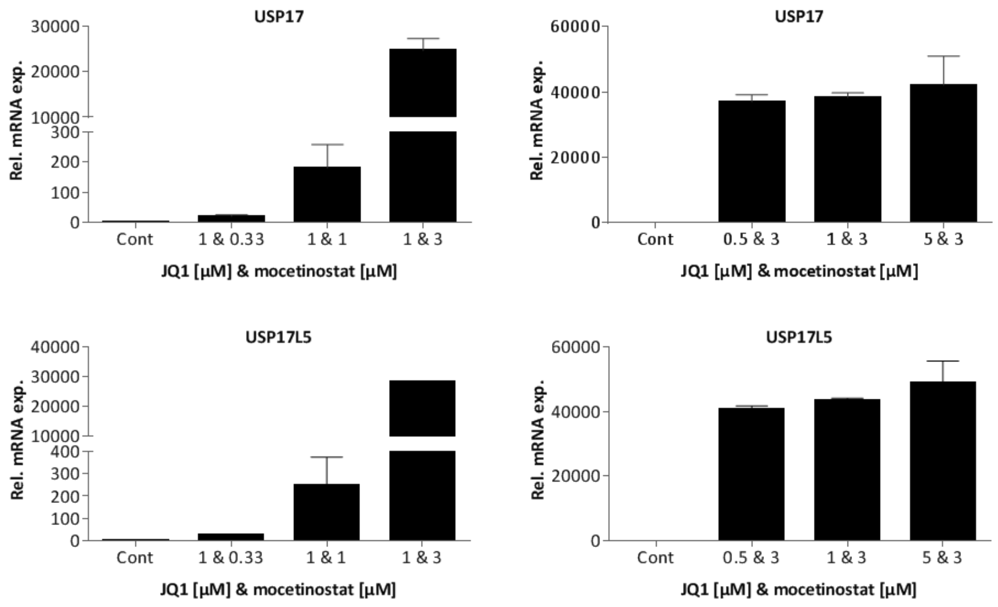

Figure S4. USP17 and USP17L5 expression in MDA-MB-231 cells after treatment with the indicated concentrations of JQ1 and mocetinostat in combination for 48 hours. USP17 and USP17L5 mRNA levels were determined by QPCR in MDA-MB-231 cells after treatment with the indicated concentrations of JQ1 and mocetinostat in combination for 48 hours. mRNA expression is shown relative to the DMSO treated (vehicle) control. Error bars represent SD from n ≥ 3 independent experiments.
